# Hypotension prediction index: comparison between invasive and non-invasive pressure inputs

**DOI:** 10.1186/s12871-025-03086-y

**Published:** 2025-04-29

**Authors:** Kamron Sarhadi, Justin Hamman, Jorge Avila, Zhongping Jian, Neal W. Fleming

**Affiliations:** 1 Department of Anesthesia & Perioperative Care, University of California, San Francisco, San Francisco, CA USA; 2 Department of Anesthesiology & Pain Medicine, University of California, Davis School of Medicine, 4150 V Street PSSB– Suite1200, Sacramento, CA 95817-1460 USA; 3 Sutter Medical Center, Sacramento, CA USA; 4 Edwards LifeSciences Critical Care, Irvine, CA USA

**Keywords:** Hypotension prediction, Non-invasive monitoring, Blood pressure, Hypotension prediction index

## Abstract

**Background:**

The Hypotension Prediction Index (HPI) derived using an Acumen™ arterial pressure transducer can decrease the incidence of intraoperative hypotension and possibly decrease perioperative complications. The HPI can also be obtained from the ClearSight™ continuous non-invasive blood pressure monitor. Concurrent comparison of HPI values obtained from these two pressure inputs is limited and additional comparisons could increase clinician confidence in non-invasive hemodynamic monitoring, expand its applications and improve patient outcomes.

**Methods:**

Simultaneous hemodynamics were recorded using two HemoSphere monitors with the HPI software and either intra-arterial Acumen^®^ (hereinafter invasive) or ClearSight™ (hereinafter non-invasive) pressure inputs. Data collected from the non-invasive system was compared to corresponding invasive, intra-arterial data using Bland-Altman analysis, Spearman correlation, concordance analysis and relative performance with respect to prediction of hypotensive events using ROC analysis and HPI alerts agreement analysis.

**Results:**

6,862 paired data points were available from 36 patients. Bland-Altman comparisons demonstrated a bias of -8.4 (± 23) with limits of agreement from − 53 to 36. The correlation between HPI values was strong with an r value of 0.76 (95%CI:0.75–0.77). Concordance was also strong at 74% (10% exclusion zone). Using ROC analysis, the AUC for prediction of hypotension was similar and at 5 min was 0.883 [0.786,0.953] for the invasive pressure and 0.860 [0.770,0.939] for the non-invasive pressure inputs. At the same time points, the agreement between HPI alerts was high with an accuracy of 86.3%.

**Conclusion:**

HPI values and predictive performance were comparable when derived from either invasive or non-invasive pressure inputs.

**Trial registration:**

The study was approved by the UC Davis Institutional Review Board (IRB #1791102-1) and registered on ClinicalTrials.gov (NCT05025176) before the enrollment of the first patient.

## Background

Intraoperative hypotension is associated with increased post-operative morbidity and mortality [1]. Hypotension is common during general anesthesia, with incidence of at least one hypotensive episode in 40% to greater than 90% of patients [2]. Intraoperative hypotension has been associated with an increased incidence of post-operative stroke, acute kidney injury, and myocardial infarction [3–5]. Continuous blood pressure measurement improves the detection of hypotension [6–8]. Direct intra-arterial pressure measurements are the standard for this measurement, but non-invasive continuous pressure can also be provided from a finger-cuff and volume-clamp technology [9, 10].

The Hypotension Prediction Index (HPI) is a newer parameter that provides advanced warning of critical hypotension [11, 12] facilitating earlier interventions, thus decreasing the incidence and magnitude of intraoperative hypotension [13]. The HPI algorithm analyzes multiple combinatorial features of the arterial pressure waveform to predict hypotension defined as a mean arterial pressure (MAP) less than 65 mmHg for at least 1 min in duration [11]. The HPI has been shown to predict hypotensive events with > 80% sensitivity and specificity [12].

The HPI was developed and evaluated using inputs from invasive, direct intra-arterial pressure measurement using the Acumen™ transducer. HPI can also be derived using continuous pressure inputs from the non-invasive ClearSight™ monitoring system. The ClearSight HPI algorithm has shown promising clinical performance, with sensitivity and specificity also exceeding 80% in a broad variety of patient populations including parturients undergoing cesarean delivery under spinal anesthesia [14], higher risk patients undergoing non-cardiac procedures under general anesthesia [15, 16] and in lower risk general surgical patients [17]. However, simultaneous comparisons of the values obtained using both inputs are limited [18]. This study was designed to obtain and compare concurrent measurements of HPI values from invasive and non-invasive pressure inputs to potentially increase confidence in non-invasive hemodynamic monitoring, expand its applications and improve patient outcomes.

## Methods

This was an observational, prospective, single-center study of adult patients undergoing surgery with general anesthesia at the University of California, Davis Medical Center in Sacramento, California. The study was approved by the UC Davis Institutional Review Board (IRB #1791102-1) and registered on ClinicalTrials.gov (NCT05025176) before the enrollment of the first patient. All activities were in adherence to the Declaration of Helsinki. Written informed consent was obtained from all participating subjects. Eligible patients were 18 years of age or older scheduled for elective surgery requiring general anesthesia with planned use of radial artery cannulation for continuous blood pressure monitoring. We excluded patients unable to provide informed consent, individuals who were not yet adults (infants, children, teenagers), pregnant women, and prisoners. No restrictions or guidelines were place on the intra-operative hemodynamic management. All available parameters from the arterial pressure transducer were displayed as per routine.

In the operating room, after induction of general anesthesia and endotracheal intubation, radial arterial cannulation was performed, and the blood pressure recorded using an Acumen™ transducer connected to a HemoSphere (Edwards Lifesciences, Irvine, CA) monitor with HPI software. A noninvasive finger cuff (ClearSight™, Edwards Lifesciences, Irvine, CA) was placed on the third or fourth digit of the same hand. The finger cuff was connected to a separate HemoSphere monitor with HPI software that was time-synchronized with the first system. All data was downloaded from the two monitors for analysis at the end of the surgical procedure.

### Comparison of 20 s interval data

All 20 s interval data sets were visually inspected and all values for spike artifacts were deleted. Multiple comparisons were made between HPI values provided from the invasive and non-invasive blood pressure monitors. Bland*-*Altman analysis was used to evaluate the agreement of the two monitoring systems. Bias was defined as the mean difference between the reference method (invasive) and the test method (non-invasive). Correlations were calculated for each pair of HPI values. Spearman r was calculated to characterize the correlation between simultaneous HPI measurements. Four-quadrant concordance analysis was also employed to assess the agreement between HPI values for invasive and non-invasive inputs. For this comparison, the percent change was calculated for consecutive measurements of HPI (at 5 min intervals) for each monitoring system, with percent change in non-invasive HPI plotted against percent change in invasive HPI to visualize their agreement. We then excluded statistical noise corresponding to changes smaller than 10%. Concordance rate was defined as the fraction of points lying in quadrants 1 (+,+) and 3 (-,-) of the 4-quadrant plot.

### Comparison of clinical performance

Continuous pressure waveform data was downloaded from both monitors and analyzed off-line to evaluate the clinical performance of the predictive algorithm. Two approaches were used for this evaluation: Receiver Operator Characteristic (ROC) analyses and HPI alerts agreement analysis.

ROC analysis has been used previously to characterize the performance of the HPI. For this analysis, we defined positive samples as data points exactly ‘t’ minutes (t = 5 or 10) prior to a hypotension event, where a hypotensive event was defined as MAP (mean arterial pressure) ≤ 65 mmHg for at least 1 min. A negative sample was selected from each non-event segment of 30-minute duration, where a non-event segment was at least 20 min from any hypotensive events and had MAP > 75 mmHg. The ROC area under the curve (AUC) along with the sensitivity, specificity, positive predictive value (PPV) and negative predictive value (NPV) at an optimal threshold with the minimum difference between sensitivity and specificity) were calculated. This analysis examines how reliably the invasive HPI was able to predict hypotensive events. The same ROC analysis was also performed for noninvasive HPI to predict hypotensive events. See Hatib et al. [11] for more details on this method of analysis for hypotension prediction.

In the HPI alerts agreement analysis, we compared the agreement between the alerts from the invasive HPI and non-invasive HPI, at the value of 85. as the current commercial implementation of HPI includes an alert at this threshold. Here, paired data was considered a true positive (TP) if non-invasive HPI was greater than or equal to 85 and the invasive system, set as the reference, also computed HPI to be greater than or equal to 85. True negative (TN) events occurred when both non-invasive and invasive HPI were less than 85. False positive (FP) events occurred when non-invasive HPI was above or equal to 85 while invasive HPI was below 85. Finally, false negative (FN) events occurred when non-invasive HPI was below 85 while invasive HPI was above or equal to 85. The whole process is summarized in Table 1. Based on these, a number of metrics can be calculated, including accuracy (%) which is the total number of true events divided by the total number of all events,, i.e., (TP + TN)/(TP + TN + FP + FN); sensitivity: TP/(TP + FN); specificity: TN/(TN + FP); positive predictive value (PPV): TP/(TP + FP), and negative predictive value (NPV): TN/(TN + FN).

**Table 1:** HPI alert agreement analysis– agreement between invasive and non-invasive HPI alerts

	Non-Invasive HPI ≥ 85	Non-Invasive HPI < 85
Invasive HPI ≥ 85	TP	FN
Invasive HPI < 85	FP	TN

TP– true positives, FN– false negatives, FP– false positive, TN– true negative

Statistical analysis for Bland-Altman, correlation and concordance was completed using GraphPad Prism version 9.2.0 for Windows, GraphPad Software, San Diego, California USA, www.graphpad.com. The D’Agostino and Pearson test was used to evaluate the distribution of all data sets. Statistics for the evaluation of clinical performance were performed with MATLAB (version R2018a; The MathWorks Inc, Natick, MA).

## Results

**Fig. 1:**
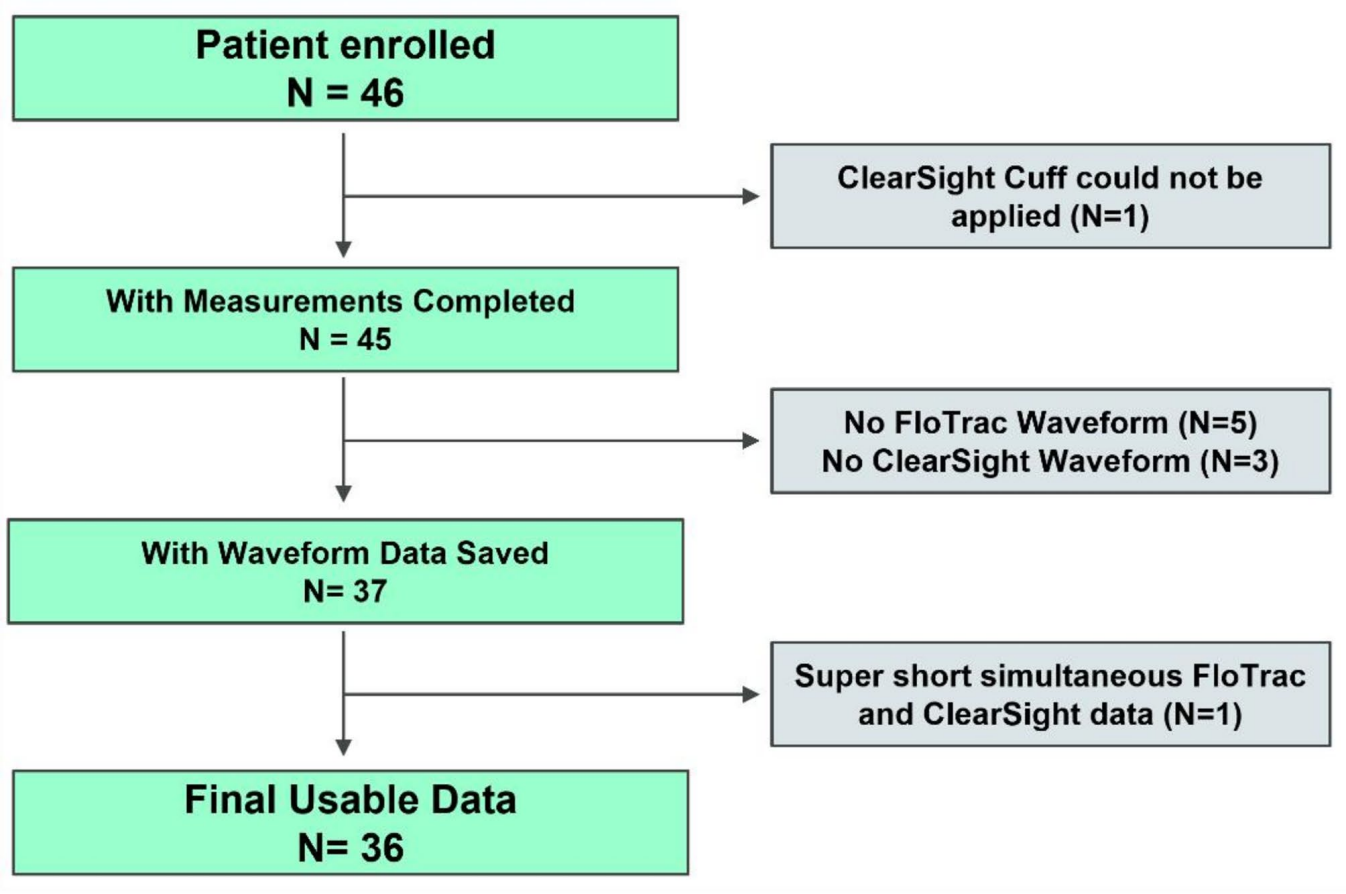
Flowchart summarizing patients included in study

46 patients provided written, informed consent and were enrolled in this study between August 26, 2021, and November 1, 2021. Sufficient data for comparison was obtained from 36 patients (Fig. 1).

Of the 36 patients studied, 21 were male, 15 were female. Their ages ranged from 28 to 92 years (average 68 ± 15.7) and body mass index ranged from 20 to 49 kg·m^2^ (average 30 ± 6.1). The surgical procedures included endovascular (16), general surgery (9), open vascular (7), orthopedic (2) and cardiac (2). Surgical case duration ranged from 59 to 748 min (average 198 ± 138.8). For the correlation analysis the number of data pairs for each patient ranged from 56 to 702 (average 191 ± 132.7). For the concordance analysis, the number of data pairs per patient ranged from 11 to 143 (average 37 ± 27.3).

**Fig. 2:**
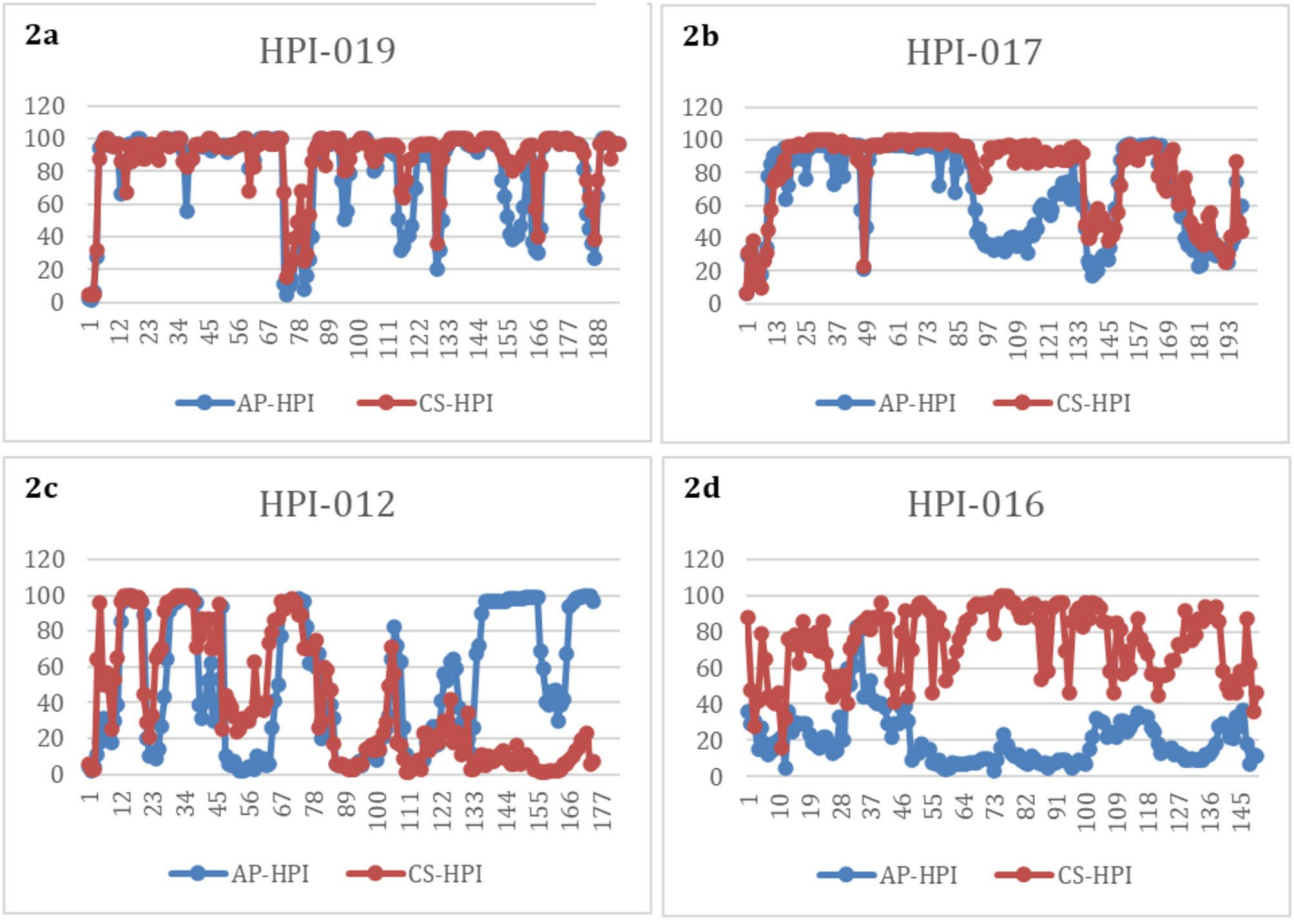
Examples of raw data showing excellent agreement (**2a**), occasional poor agreement (**2b**, **2c**), and poor agreement throughout (**2d**). AP-HPI–invasive arterial pressure input, CS-HPI–noninvasive arterial pressure input

By visual inspection, the agreement between invasive and non-invasive HPI values was often excellent, but sometimes varied during the procedure and occasionally was poor (Fig. 2).

**Fig. 3:**
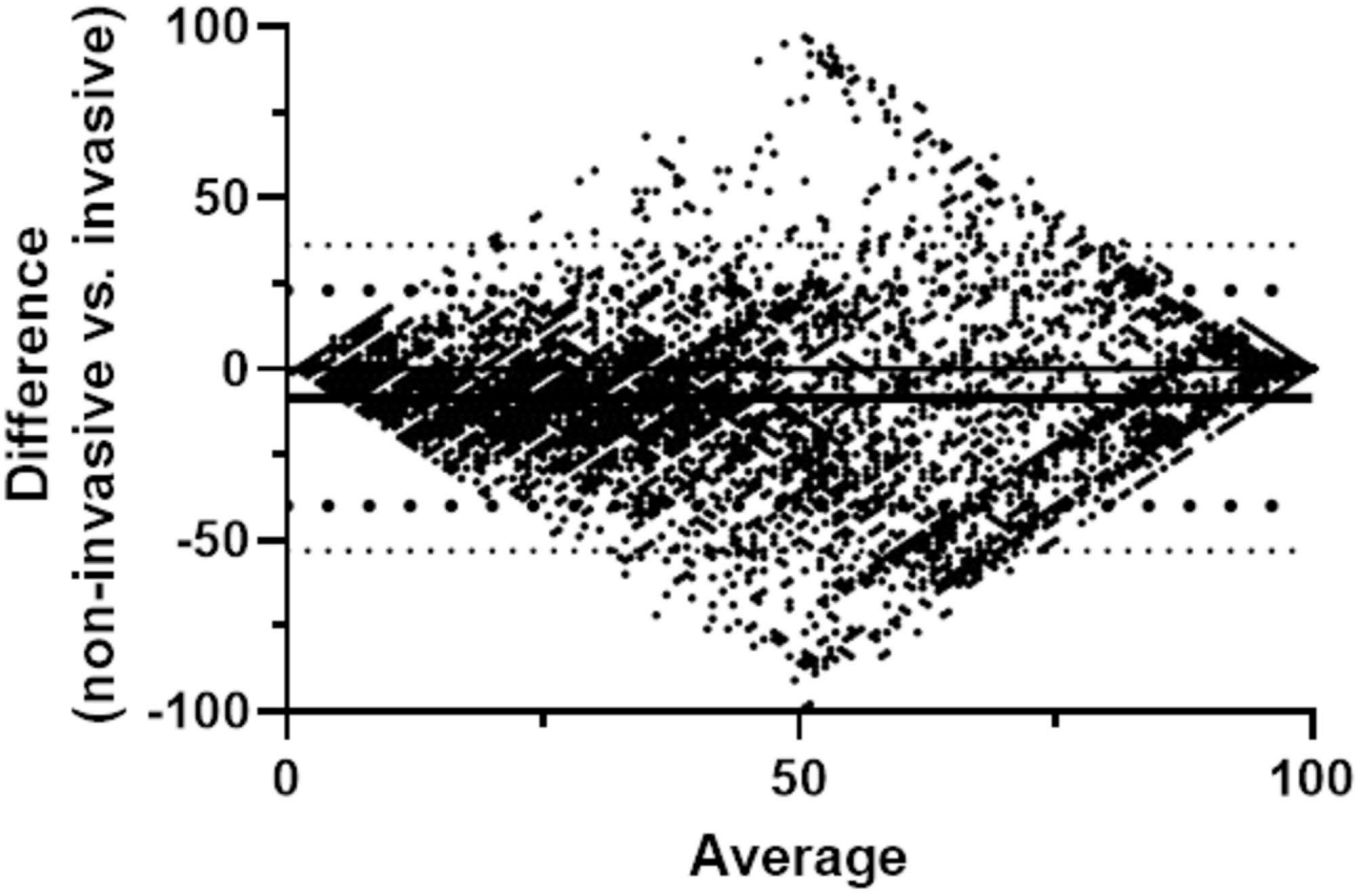
Bland-Altman analysis for non-invasive HPI in comparison to invasive HPI

For Bland-Altman and correlation comparisons, a total of 6,862 data pairs were available for analysis. Bland-Altman analysis for the non-invasive HPI, in comparison to invasive HPI, demonstrated a bias of -8.4 ± 23 with 95% limits of agreement of -53, 36 (Fig. 3).

**Fig. 4:**
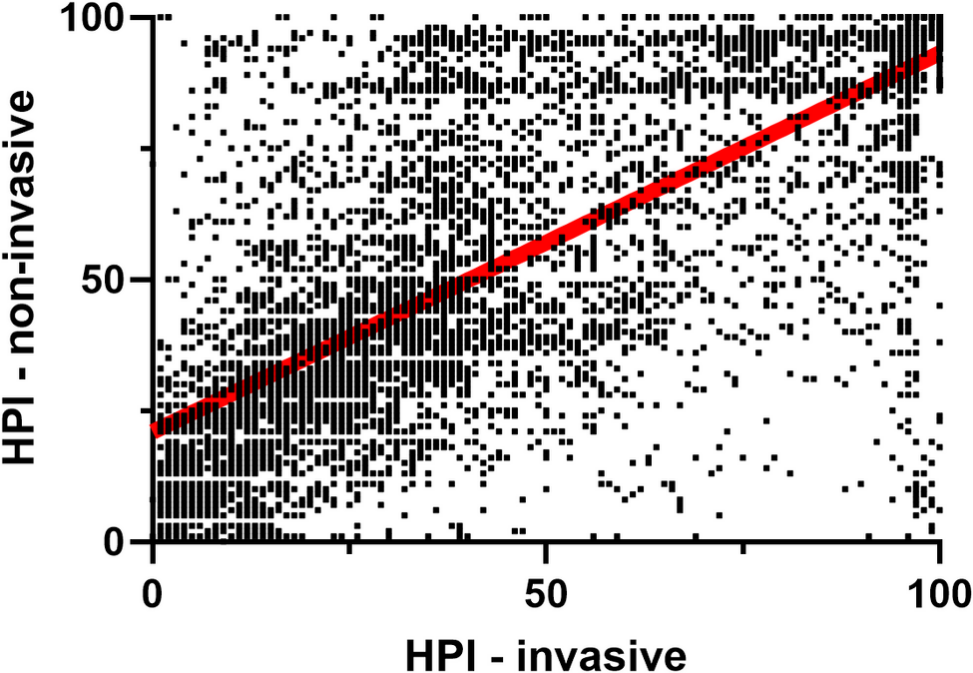
Correlation between HPI values from invasive and non-invasive arterial pressure inputs with regression line (red)

For the correlation analysis, the HPI values were not normally distributed, so we computed the nonparametric Spearman correlation (*r* = 0.76, 95%CI 0.75, 0.77) (Fig. 4).

**Fig. 5:**
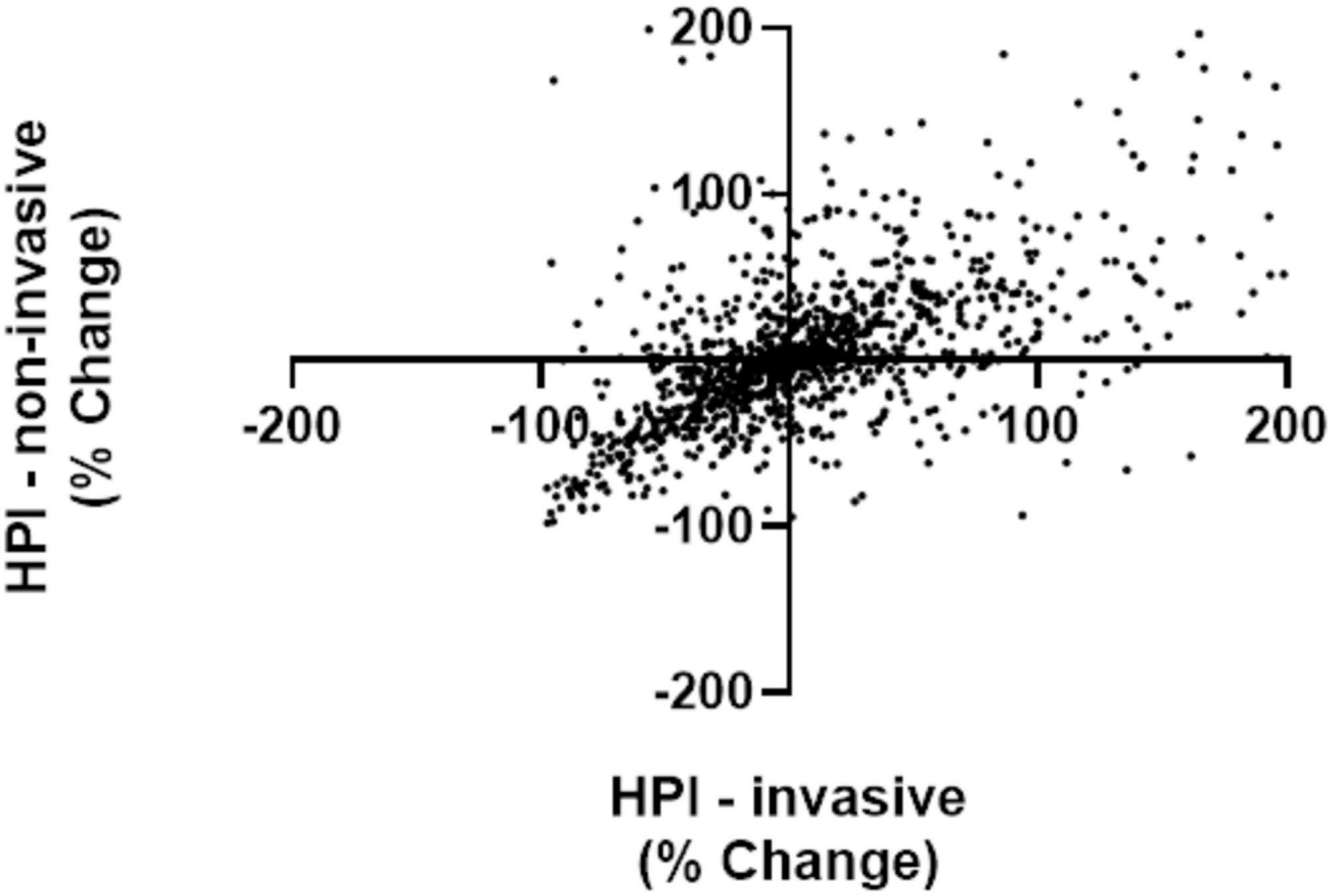
Concordance analysis between invasive HPI and non-invasive HPI

The concordance analysis comparisons were made with respect to the relative changes over 5-minute intervals (Fig. 5). A total of 1,328 data pairs were therefore available for this analysis. We evaluated exclusion zones of 0, 5, 10 and 15%. Increasing the exclusion zone had no significant impact on the concordance rates of 75.0, 73.8, 73.8 and 73.7%, respectively.

**Fig. 6:**
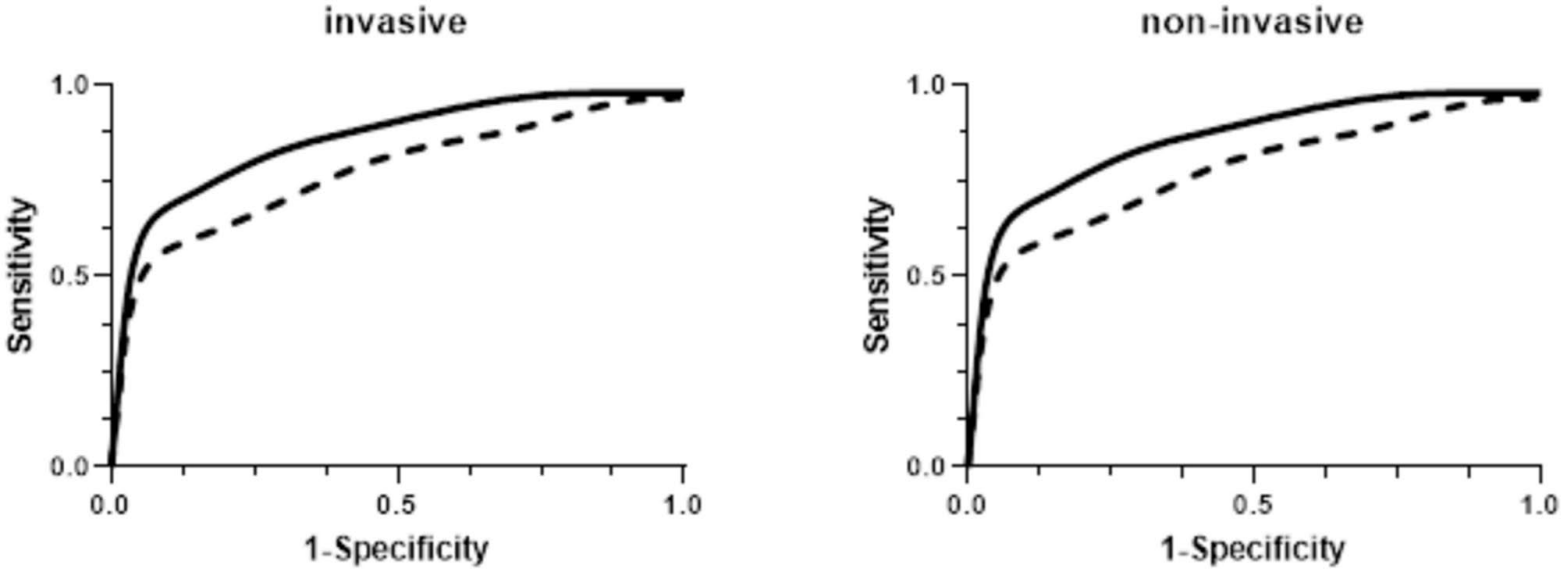
ROC analysis curves for invasive and non-invasive blood pressure inputs at -5 minutes ( ⎯ ) and -10 minutes ( - - )

To assess clinical performance, we performed ROC analysis for both the invasive and non-invasive systems at 5 and 10 min prior to the onset of a hypotensive event and compared the Positive and Negative Predictive Values for both pressure inputs. For the ROC analysis, the Area Under Curve (AUC) measurements for invasive blood pressure inputs were 0.88 [0.79, 0.95] and 0.80 [0.63, 0.92] at -5 and − 10 min, respectively. For non-invasive blood pressure inputs, the AUC measurements were 0.86 [0.77, 0.94] and 0.76 [0.60, 0.89] at -5 and − 10 min, respectively (Fig. 6). Sensitivity and specificity results were also comparable for the two inputs and are presented in Table 2.

The positive predictive values for the invasive pressure inputs were 0.86 [0.71, 0.96] and 0.70 [0.48, 0.87] at -5 and − 10 min, respectively. For the non-invasive pressure inputs the PPV values were 0.83 [0.66, 0.95] and 0.70 [0.44, 0.90] at -5 and − 10 min, respectively. The negative predictive values for the invasive pressure inputs were 0.69 [0.39, 0.89] and 0.72 [0.46, 0.90] at -5 and − 10 min, respectively. For the non-invasive pressure inputs the NPV values were 0.70 [0.45, 0.89] and 0.68 [0.44, 0.85] at -5 and − 10 min, respectively. All clinical performance values are presented in Table 2.

**Table 2:** ROC Analysis– invasive and non-invasive HPI inputs to predict hypotension and corresponding positive and negative predictive value calculations

Time	AUC	Sensitivity	Specificity	PPV	NPV	Threshold
-5 min (invasive HPI)	0.88 [0.70,0.95]	0.79 [0.65,0.91]	0.79 [0.64,0.91]	0.86 [0.71,0.96]	0.69 [0.39,0.89]	34
-5 min (non-invasive HPI)	0.86 [0.77,0.94]	0.78 [0.68,0.87]	0.78 [0.68,0.88]	0.83 [0.66,0.95]	0.70 [0.45,0.89]	40
-10 min (invasive HPI)	0.80 [0.63,0.92]	0.71 [0.55,0.85]	0.71 [0.55,0.85]	0.70 [0.48,0.87]	0.72 [0.46,0.90]	28
-10 min (non-invasive HPI)	0.76 [0.60,0.89]	0.70 [0.56,0.83]	0.70 [0.55,0.83]	0.70 [0.44,0.90]	0.68 [0.44,0.85]	37

AUC– Area under curve, PPV– Positive predictive value. NPV– Negative predictive value. Threshold– Minimal sensitivity/specificity difference. All values are presented as mean [95% CI]

The HPI alerts agreement analysis showed an accuracy of agreement between invasive HPI alerts and non-invasive HPI alerts [(TP + TN)/(TP + TN + FP + TN)] of 86.3%, sensitivity [TP/(TP + FN)] of 81.0%, specificity [TN/(TN + FP)] of 87.5%, PPV [TP/(TP + FP)] of 60.9%, and NPV [TN/(TN + FN)] of 95.1%.

## Discussion

Intraoperative hypotension is a common clinical occurrence that is associated with an increased incidence of multiple postoperative complications in many surgical patient populations [1–5]. Continuous blood pressure monitoring increases the incidence and shortens the time to detection of hypotension [6–8]. Arterial cannulation for continuous pressure monitoring is the gold standard, but it is invasive and there are associated risks and complications that limit its use. Noninvasive alternatives for continuous blood pressure measurement are available with the potential to increase the use of continuous pressure monitoring and extend its advantages to a broader patient population [9, 10]. 

A second opportunity to decrease the incidence of intraoperative hypotension is the Hypotension Prediction Index (HPI). The HPI is the result of a machine learning algorithm that estimates the likelihood of an oncoming hypotensive event [11]. The HPI algorithm uses continuous arterial pressure waveform data as the input, extracts various waveform features, then together with the patient demographics (age, gender, height, and weight), computes an index value that ranges between 0 and 100. The larger the HPI value, the more likely and the sooner a hypotensive event will occur [12]. There is currently an active debate in the literature regarding the comparative predictive value of HPI and MAP [19, 20]. However, our study was designed to compare HPI values generated with two different pressure inputs, a question that is distinct from the HPI performance in comparison to other parameters.

The initial development and demonstrated utility of the HPI used blood pressure inputs from arterial catheters. Subsequently the HPI algorithm was applied to blood pressure measurements obtained with the noninvasive ClearSight monitoring system. Initial assessments of the clinical efficacy of the ClearSight Hypotension Prediction Index (HPI) have yielded outcomes comparable to those obtained with invasive arterial pressure inputs across diverse patient cohorts. Frassanito et al. conducted a retrospective analysis examining the performance of ClearSight HPI in 50 pregnant patients undergoing cesarean delivery with spinal anesthesia [14]. They reported sensitivity and specificity exceeding 80% for predicting hypotensive events 3 min prior to occurrence. In second investigation by Frassanito et al. of 28 patients undergoing gynecologic oncologic surgery [15], the ClearSight HPI demonstrated similar sensitivity and specificity of over 80% for predicting hypotensive events up to 15 min prior to their occurrence. Maheshwari et al. retrospectively analyzed data from 320 patients classified as American Society of Anesthesiologists (ASA) physical status 3 or 4 undergoing moderate to high-risk non-cardiac procedures [16]. They found sensitivities and specificities of 75% at 15 min and 83% 10 min before hypotensive events, affirming the utility of ClearSight HPI in preemptively identifying hypotension in clinically challenging scenarios. Wijnberge et al. extended this observation to a similarly large (507 patients) lower risk general surgical population [17]. They demonstrated PPVs of 88% and 79%, respectively, 5 and 10 min prior to a hypotensive event. Rellum et al. retrospectively analyzed HPI values concurrently collected from 80 ASA 2 general surgical patients [18]. They reported comparable AUC values for the ROC curves for hypotension prediction for invasive and non-invasive pressure inputs. However, they also commented that small differences in the arterial pressure waveform morphology could impact this index in unknown ways. This concurrent comparison of HPI performance was designed to explore this possibility.

Review of the sample trend graphs for HPI over time illustrates some of the difficulties in head-to-head comparisons of HPI values. HPI values may change rapidly, especially in middle ranges, a small difference in response time can produce large differences in the absolute values that are not clinically relevant. This characteristic has significant impacts on standard methodologies for comparisons of measurements. In the Bland-Altman comparison this is manifested by the marked expanded differences in the values in the middle range of the HPI index and the consequent wide limits of agreement. (It should be noted that this analysis was not adjusted for repeated measures. Consequently, the limits of agreement reported may be wider, with larger confidence intervals, but the limitations of this approach to comparative analysis remain.) In the correlation analysis, this scatter shifts the best fit line. If the linear regression line is constrained to include the origin, it becomes indistinguishable from the line of identity (slope = 1.03 [1.02, 1.04]. The impact of any small response time difference is buffered by the 4-quadrant correlation analysis but the required smoothing to 5-minute intervals will also not capture any rapid changes with either input. Given these limitations of standard comparison techniques, we also compared clinical performance characterizations of the simultaneously collected HPI values. ROC analysis of the clinical performance of the HPI values provided nearly identical values for AUC at both 5 and 10 min prior to a hypotensive event along with nearly identical sensitivity and specificity calculations. Similarly, both the positive and negative predictive values for HPI were nearly identical for the two pressure inputs. In addition, since the current commercial implementation of HPI includes an alert at the value of 85, we compared the agreement between HPI alerts and analyses showed that the two alerts agree with each other quite well, with an accuracy of 86.3%.

Our study demonstrates that the HPI derived from non-invasive finger-cuff ClearSight monitor is reliable, as it provides values with predictive performance that are comparable to the gold standard invasive arterial catheter for hemodynamic monitoring. This supports the use of HPI for a wider array of surgeries where an invasive monitor is not planned or indicated. However, some limitations of this data should be highlighted. The study population was limited to higher risk patients and procedures in whom invasive arterial pressure monitoring was part of the planned operative management. Extrapolation of these conclusions to other patient populations should be done cautiously. Second, this was a relatively small sample size. Evaluation of the clinical performance of the HPI algorithm with non-invasive pressure input should be validated in a larger patient population with focused data collected for this outcome. Lastly, it should be noted that even in this small sample population there was one patient in whom the non-invasive ClearSight cuff could not be applied due to arthritic contractures of the fingers in both hands.

Overall, the predictive clinical performance of HPI derived from the non-invasive ClearSight finger cuff compares well to that obtained from the gold standard invasive arterial pressure catheter used for hemodynamic monitoring. This supports the broader use of both continuous, non-invasive pressure monitoring and the HPI parameter to predict and prevent hypotensive episodes which may result in better patient outcomes.

## Data Availability

The data sets used and/or analyzed during the current study are available from the corresponding author on reasonable request.

## Abbreviations

ASA: American society of anesthesiologists
AUC: Area under the curve
HPI: Hypotension prediction index
FN: False negative
FP: False positive
MAP: Mean arterial pressure
NPV: Negative predictive value
PPV: Positive predictive value
ROC: Receiver operator characteristic
TN: True negative
TP: True positive
